# Parent-Adolescent Communication and Early Adolescent Depressive Symptoms: The Roles of Gender and Adolescents’ Age

**DOI:** 10.3389/fpsyg.2021.647596

**Published:** 2021-05-10

**Authors:** Qiongwen Zhang, Yangu Pan, Lei Zhang, Hang Lu

**Affiliations:** ^1^Research Institute of Social Development, Southwestern University of Finance and Economics, Chengdu, China; ^2^The School of Law, Chengdu University, Chengdu, China; ^3^School of Law, Southwest Petroleum University, Chengdu, China

**Keywords:** father-adolescent communication, mother-adolescent communication, early adolescent depressive symptoms, gender, age

## Abstract

Positive parent-adolescent communication has been found to be negatively related to adolescent depressive symptoms; however, few studies have investigated the moderating effects of adolescent gender and age on this relationship, especially during early adolescence in China. The present study investigated the joint moderating effects of adolescent gender and age on the linkage of father-adolescent and mother-adolescent communication with adolescents’ depressive symptoms. A total of 11,455 Chinese junior high school students (*M*_*age*_ = 14.15 years, *SD* = 1.22 years; 49.86% boys; *N*_*grade*7_ = 5712, *N*_*grade*9_ = 5743) completed *ad hoc* questionnaires of parent-adolescent communication and depressive symptoms. Multiple linear regression analyses were conducted. Results indicated that gender and age jointly moderated the association between parental communication and adolescent depressive symptoms. Specifically, for girls, the negative effects of both father-adolescent and mother-adolescent communication on depressive symptoms were stronger in 9th grade students than in 7th grade students, while for boys, the negative effects were not different between 7th grade students and 9th grade students. These findings suggest that in China, the protective effects of positive parent-adolescent communication on adolescents’ depressive symptoms may be most salient among senior-grade girls in junior high school.

## Introduction

Depression is a serious mental health problem that has a detrimental effect on adolescents’ psychosocial functioning (Rawana and Morgan, 2014; Alaie et al., 2021). Depression in adolescents has been found to be associated with many negative outcomes, such as a higher risk of suicide (Weissman et al., 1999; Hovanesian et al., 2009), poorer academic performance (Verboom et al., 2013), and higher levels of substance abuse (Obando et al., 2004; Scholes-Balog et al., 2015). Early adolescents are vulnerable to depression (Cole et al., 2002; Solomon-Krakus et al., 2017), because early adolescence is an important transition period from childhood to adolescence during which adolescents face greater challenges and stressors from all the tasks of physical, psychological, and social development (Cicchetti and Rogosch, 2002; Hammen, 2009). According to the 2017 National Survey on Drug Use and Health, 13.3% of adolescents aged between 12 and 17 suffered a major depressive episode in the United States in 2017 (National Institute of Mental Health, 2019). In particular, it has been reported that early adolescents in China experience more stress, such as increased learning burden, high expectations from parents, and a weakened parent-adolescent bond, than adolescents in Western countries (Sun et al., 2012). Recently, a meta-analysis indicated that the prevalence of depressive symptoms among adolescents in secondary schools in mainland China was 24.3% (Tang et al., 2019). As depressive symptoms have severe and adverse consequences on early adolescents’ psychosocial adjustment in China, it is essential to identify protective factors to inform the development of intervention and prevention efforts. Cicchetti and Rogosch (2002) suggested that we should consider depressive symptoms among early adolescents in dynamic relation between individual and internal and external contexts.

### Parent-Adolescent Communication and Adolescent Depressive Symptoms

Positive parent-adolescent communication is an important protective factor in preventing adolescents’ depressive symptoms (Ioffe et al., 2020; Kapetanovic et al., 2020). The high quality of parent-adolescent communication can strengthen parental connectedness, intimacy, trust, family cohesion, and family adaptability (Barnes and Olson, 1985; Laursen and Collins, 2004; Cava et al., 2014) and provide emotional and instrumental support for children, which has been found to be associated with low levels of internalizing and externalizing problems in adolescents (Hamza and Willoughby, 2011; Garthe et al., 2015; Ioffe et al., 2020). On the contrary, low-quality parent-adolescent communication, such as parental rejection, parental criticism, adolescents’ secrecy, and non-disclosure can contribute to the development of depressive symptoms (Hale et al., 2005; Frijns et al., 2010). Moreover, a meta-analysis of 164 articles demonstrated that poor parent-adolescent communication was the strongest among fifteen psychosocial risk factors for depressive symptoms among adolescents in secondary schools in mainland China (Tang et al., 2020).

Attachment theory provides us with a developmental perspective to understand the effect of parent-adolescent attachment on depressive symptoms during early adolescence (Duchesne and Ratelle, 2014). Bowlby (1982) suggested that the bidirectional interaction between infant and caregiver will develop an internal working model of social relationships, i.e., parental attachment, which is the foundation of child emotional and social development (Steinberg and Morris, 2001; Brumariu, 2015) and has been associated with psychological outcomes, such as depressive symptoms and externalizing behavior (Allen et al., 2007). Studies have shown that adolescents with insecure attachment had more depressive symptoms (Agerup et al., 2015; Rawatlal et al., 2015; Cortés-García et al., 2019). In addition, the relationship between insecure attachment and depression was mediated by cognitive-emotional factors, such as brooding rumination, self-criticism, and low self-compassion (Cortés-García et al., 2020). Conversely, adolescents with secure attachment develop emotion regulation abilities in challenging situations through repeated positive interactions with their parents, who encourage adolescents to manage negative emotions and communicate openly about their emotional state to their parents so that they can provide timely support to their child (Parrigon et al., 2015; Allen and Tan, 2016). Further, securely attached adolescents were more likely to gain communication and perspective-taking skills to coordinate parent-adolescent discrepancies in autonomy and solve parental conflicts, so they were able to achieve success in gaining autonomy while maintaining relatedness with their parents (Allen et al., 2007). In contrast, insecurely attached adolescents reported more parental criticism (Anhalt and Morris, 2008) and resisted discussing negative events that may activate the attachment system due to poor parent-adolescent communication (Allen and Tan, 2016).

Some studies have investigated parent-adolescent communication in Chinese adolescents compared to that of Western adolescents. For example, Chinese adolescents reported lower quality parent-adolescent communication compared to Italian adolescents (Li et al., 2015). Chinese parents were less willing to express their verbal, non-verbal, and supportive affection to their children relative to parents in the United States (Zhang and Wills, 2016). Moreover, Chinese parents showed their love to children mainly through instrumental support, but not open communication, while American parents emphasized open communication to increase parental warmth (Wu and Chao, 2011). In Chinese culture, adolescents are expected to be obedient, exhibit self-control, and respect their parents, while parents as authoritarian figures are inclined to give guidance to their children on conduct and morality (Wu et al., 2002; Liu et al., 2005; Chuang and Su, 2009). When adolescents disagree with their parents, they are less likely to openly express their own conflicting ideas. In fact, parents’ awareness of their children’s information relies on adolescents’ self-disclosure during adolescence (Keijsers et al., 2009). If parents know little information about their children, they cannot provide timely support; as a result, adolescents tend to develop depressive symptoms (Pantaleao and Ohannessian, 2019). Moreover, as good academic performance is valued highly in Chinese culture, Chinese adolescents face higher levels of academic stress compared with Western adolescents, especially for students in grade 9 who are facing the senior high school entrance examination (Sun et al., 2012). Studies have shown that when academic achievement was the core content of parent-adolescent communication, adolescents with poor academic performance were more likely to be depressed (Ma et al., 2018; Tang et al., 2020).

### Different Effects of Father-Adolescent Communication and Mother-Adolescent Communication on Adolescents’ Depressive Symptoms

Father-adolescent communication and mother-adolescent communication have different effects on adolescents’ depressive symptoms (Ioffe et al., 2020). According to attachment theory, children report greater *safe haven* support from mothers in times of distress as well as greater *secure base* support from fathers from which to explore, suggesting that mothers and fathers play different roles in children’s secure attachment (Kerns et al., 2015). As mothers typically are in charge of daily care and emotional comfort for their children (Ho, 1987; Shek, 1999), they are inclined to communicate more actively, emotionally, and frequently with their children (Barnes and Olson, 1985; Shek, 2000). Relative to fathers, mothers may receive more information about their children’s problems and provide timely support and care, which can relieve children’s depressive symptoms (Darling et al., 2006; Pantaleao and Ohannessian, 2019). However, as the role of fathers in children’s development has received increased research attention (Flouri and Buchanan, 2003; Dubeau et al., 2013; Paquette et al., 2013), father-adolescent communication has more recently become an area of investigation. For example, Ioffe et al. (2020) demonstrated that paternal communication had a greater impact on children’s internalizing symptoms than maternal communication. Further, Lopez et al. (2005) found that negative father-adolescent communication increased the risk of victimization that is linked with depressive symptoms. Relative to mothers, fathers can provide more problem-solving strategies and autonomy support that are useful for their children (Lamb and Lewis, 2013; Huang et al., 2021).

### Moderating Effect of Gender on the Relation Between Parent-Communication and Adolescents’ Depressive Symptoms

Adolescents’ gender could moderate the relationship between parent-adolescent communication and adolescents’ depressive symptoms, with stronger associations observed for female adolescents (Finan et al., 2018; Pantaleao and Ohannessian, 2019). Studies have shown that female adolescents communicated more often with their mothers than male adolescents, and were more satisfied with communication with mothers than with fathers (Noller and Bagi, 1985; Noller and Callan, 1990). Moreover, girls tended to express greater closeness and shared more personal issues with their mothers than with their fathers (Collins and Russell, 1991; Van Lissa et al., 2019), and mothers provided various resources, such as emotional comfort and social support, to decrease the level of depressive symptoms (Katz and Hunter, 2007; Kenny et al., 2013). Thus, female adolescents may be more likely to be affected by maternal relationships than paternal relationships (Rueger et al., 2014). In addition, a previous study revealed that father-adolescent communication predicted depressive symptoms in boys, but not in girls (Pantaleao and Ohannessian, 2019). Relative to female adolescents, male adolescents perceived more positive communication with their fathers and may receive more support that can reduce their depressive symptoms (Jackson et al., 1998; Cornwell, 2003).

### Moderating Effect of Adolescents’ Age on the Relation Between Parent-Adolescent Communication and Adolescents’ Depressive Symptoms

Age of the adolescent is another factor that could influence the relation between parent-adolescent communication and adolescents’ depressive symptoms. With the development of autonomy and individuality, adolescents are more likely to keep secrets from their parents, which is associated with depressive symptoms, especially when adolescents consider parental solicitation as intrusion and control (Kakihara et al., 2010). Some empirical evidence has shown that the effect of parental communication on adolescent psychological outcomes changes with age. For example, Xu et al. (2016) found that, relative to Chinese adolescents in grade 7, Chinese adolescents in grade 9 developed more self-consciousness, idealized their parents less, and had reduced communication with their parents. As such, parents cannot provide social support because they have less information about their children, which increases the level of depressive symptoms. However, Hamza and Willoughby (2011) found that adolescent disclosure had a negative impact on depressive symptoms indirectly through parents’ knowledge, but the effect did not change with age across high school years. Few studies have investigated the moderating effect of adolescents’ age on the relationship between parent-adolescent communication and adolescents’ depressive symptoms.

### The Present Study

As early adolescence is a key developmental stage characterized by complex biological, social, and psychological changes as well as various stresses, it is necessary to understand the role of parent-adolescent communication as a critical dimension of parent-adolescent attachment in early adolescent mental health (Armsden and Greenberg, 1987; Allen and Tan, 2016). Although previous studies have found that father-adolescent and mother-adolescent communication influences adolescents’ depressive symptoms, few studies have investigated the combined moderating effects of adolescents’ age and gender on the relationship between father-adolescent or mother-adolescent communication and adolescents’ depressive symptoms during early adolescence in China. Therefore, the current study aimed to answer three questions. First, do parent-adolescent communication and adolescents’ depressive symptoms change during early adolescence in China? Second, does parent-adolescent communication influence Chinese early adolescents’ depressive symptoms? Third, do adolescents’ age and gender jointly moderate the effects of parent-adolescent communication on Chinese early adolescents’ depressive symptoms? Based on the literature reviewed above, the present study proposed three hypotheses:

H1 Relative to 7th grade students, the quality of both father-adolescent communication and mother-adolescent communication would be lower, while adolescents’ depressive symptoms would be higher among 9th grade students.
H2 Both positive father-adolescent communication and mother-adolescent communication would have a negative effect on early adolescent depressive symptoms.
H3 Adolescents’ age and gender would jointly moderate the effect of parent-adolescent communication on early adolescents’ depressive symptoms. Specifically, positive father-adolescent or mother-adolescent communication would have a stronger effect on female adolescents’ depressive symptoms in 9th grade students than in 7th grade students, while positive father-adolescent or mother-adolescent communication would have a weaker effect on male adolescents’ depressive symptoms in 9th grade students than in 7th grade students.

## Materials and Methods

### Participants and Procedure

The data used in this study were obtained from the China Education Panel Survey, which was conducted by the National Survey Research Center at Renmin University of China, using a multi-stage sampling method with probability proportional to size. First, they sampled 28 counties (districts) from all 2870 counties in China. Second, they sampled four schools from each selected county by using probability proportionate to size sampling. Third, in each selected school, they randomly sampled two classes from each of grades 7 and 9. Finally, all students in the selected classes participated in the survey. Between September 2013 and March 2014, a total of 19,487 students (including 10,279 in grade 7 and 9208 in grade 9) from 112 schools and 438 classes participated in the survey. Participants were asked to complete a self-report questionnaire in class at school under the guidance of trained investigators.

In the present study, given that we investigated the effects of both father- and mother-adolescent communication on adolescents’ depressive symptoms, we selected participants whose fathers and mothers were both at home, and whose Hukou (a legal document that records the household population’s basic information) was local. As a result, there were 12,018 participants. Among the 12,018 participants, 399 participants (3.3%) had no data on depressive symptoms. Additionally, given that skewness = 1.04 and kurtosis = 1.51 on depressive symptoms, we deleted 164 participants whose depressive symptoms score was the maximal value (151 participants scored 25 and 13 participants scored 24). Finally, there were 11,455 participants included in the data analysis (*M*_*age*_ = 14.15 years, *SD* = 1.22 years; *N*_*boys*_ = 5,711, *N*_*girls*_ = 5,744; *N*_*grade*7_ = 5,712, *N*_*grade*9_ = 5,743). Among these 11,455 participants, 670 participants (5.8%) had no data on father-adolescent communication and 257 participants (2.2%) had no data on mother-adolescent communication. Considering the low missing ratio and the random missing data pattern, we deleted the participants with missing data on relevant variables when conducting the data analysis.

### Measures

#### Father-Adolescent and Mother-Adolescent Communication

According to the measurement of parental solicitation, as a main component of parent-driven communication (Stattin and Kerr, 2000; Kapetanovic et al., 2020), we used five items to measure father-adolescent communication. Participants were asked, “How often did your father have a discussion with you on the following issues?” The five response items were, “What happened at school,” “Your relationship with your friends,” “Your relationship with your teacher,” “Your feelings,” and “Your mind or troubles.” We assessed mother-adolescent communication using the same five items, except they were asked, “How often did your mother have a discussion with you on the following issues?” The items were responded to using a three-point scale where 1 = “never,” 2 = “once in a while,” and 3 = “often.” In this study, Cronbach’s α for father-adolescent communication was 0.84, and for mother-adolescent communication α = 0.82.

#### Depressive Symptoms

According to the definition and measurement of depressive symptoms (Radloff, 1977; Saylor et al., 1984), we used five items to measure depressive symptoms. Participants were asked, “Have you had any of the following feelings in the past 7 days?” The five items were, “upset,” “depressed,” “unhappy,” “life has no meaning,” and “sad.” The items were responded to using a five-point scale ranging from 1 = “almost never” to 5 = “almost always.” Cronbach’s α in this study was 0.86.

#### Demographics Variables

We collected demographic data on participants’ gender, grade, family economic condition, and parents’ education level. We used one item to measure the family’s economic condition, namely, “What is your family’s financial situation at present?” The item was responded to using a three-point scale ranging from 1 = “very poor,” 2 = “middle income,” and 3 = “very rich.” Parents’ education level was assessed by two items, “What is your mother’s education level?” and “What is your father’s education level?” Participants selected one from nine levels, including “No education whatsoever,” “Primary school,” “Junior high school,” “Technical secondary school,” “Vocational high school,” “High school,” “University college,” “University degree,” and “Postgraduate or above.” Additionally, we collected data on two variables at the school level: school rank (three categories, including “Medium and below,” “Above average,” and “Best”) and school location (three categories, including “Central urban area,” “Urban-rural fringe area,” and “Town and country”).

### Data Analysis Strategy

First, we examined whether the variables in the study were normally distributed by determining skewness and kurtosis. Second, we analyzed the means, standard deviations, and bivariate correlations of all variables. Third, we examined grade and gender differences in the average level of parent-adolescent communication and depressive symptoms using *t*-tests. Fourth, we used multiple regression to examine the effects of father-adolescent and mother-adolescent communication on adolescents’ depressive symptoms in the total sample. Finally, we used multiple regression to investigate the moderating effect of adolescents’ gender on the moderating effect of grade on the relationship between father-adolescent or mother-adolescent communication and adolescents’ depressive symptoms. In the multiple regressions, we controlled for school rank, school location, family economic status, father’s education level, and mother’s education level. Although the data had a multilevel structure, we did not use multilevel regression analyses because the intra-class correlation coefficient (ICC) of the dependent variable was 0.040 [i.e., it did not reach 0.059 (Cohen, 1988)]. All analyses were performed using SPSS 21.0. In particular, data analysis for the moderating effect of adolescents’ gender on the moderating effect of grade on the relationship were conducted using Model 3 in the Process macro for SPSS Statistics (Hayes, 2016).

## Results

### Normal Distribution Test and Sample Distribution of the Categorical Variables

The results of the test for the normal distribution of the variables were as follows: skewness = 0.68 and kurtosis = 0.30 for depressive symptoms, skewness = 0.08 and kurtosis = −0.82 for father-adolescent communication, and skewness = −0.23 and kurtosis = −0.79 for mother-adolescent communication. These results indicated that these variables were normally distributed. The results of the sample distribution on the categorical variables are displayed in Table 1.

**TABLE 1:** Sample distribution of school rank, school location, family economic status, grade, gender, and father and mother education.

Variable	*n*	%	Variable	*n*	%
**School rank**			**Father education**		
Medium and below	1, 852	16.2	No education whatsoever	46	0.4
Above average	6, 646	58.0	Primary school	1, 468	12.8
Best	2, 957	25.8	Junior high school	4, 780	41.7
**School location**			Technical secondary school	744	6.5
Central urban area	4, 744	41.4	Vocational high school	258	2.3
Urban-rural fringe area	2, 651	23.1	High school	2, 021	17.6
Town and country	4, 060	35.4	University college	838	7.3
**Family economic status**			University degree	1, 090	9.5
Poor	2, 114	18.5	Postgraduate or above	197	1.7
Middle income	8, 581	74.9	Missing	13	0.1
Rich	738	6.4	**Mother education**		
Missing	22	0.2	No education whatsoever	320	2.8
**Grade**			Primary school	2, 032	17.7
Grade 7	5, 712	49.9	Junior high school	4, 654	40.6
Grade 9	5, 743	50.1	Technical secondary school	757	6.6
**Gender**			Vocational high school	248	2.2
Boys	5, 711	49.9	High school	1, 623	14.2
Girls	5, 744	50.1	University college	771	6.7
			University degree	919	8.0
			Postgraduate or above	118	1.0
			Missing	13	0.1

**N* = 11,455.*

### Age and Gender Differences in Parent-Adolescent Communication and Depressive Symptoms

Results of the *t*-tests on age and gender differences are provided in Tables 2–4. In this study, age was represented by grade; generally, participants in Grade 9 were 2 years older than participants in Grade 7 (*M*_*age*_ = 13.14 years in Grade 7 vs. *M*_*age*_ = 15.15 years in Grade 9). Participants in Grade 9 had less father-adolescent communication (*M*_*grade*9_ = 9.45 vs. *M*_*grade*7_ = 9.88) and less mother-adolescent communication (*M*_*grade*9_ = 10.75 vs. *M*_*grade*7_ = 11.06), and had more depressive symptoms (*M*_*grade* 9_ = 10.48 vs. *M*_*grade* 7_ = 9.59) compared to those in Grade 7. Additionally, collapsed across grades, girls reported more mother-adolescent communication (*M*_*girls*_ = 11.28 vs. *M*_*boys*_ = 10.51) and more depressive symptoms (*M*_*girls*_ = 10.26 vs. *M*_*boys*_ = 9.82) than boys.

**TABLE 2:** Means and standard deviations of parent-adolescent communication and depressive symptoms according grade.

Variable	Grade 7	Grade 9	*t*	*p*	Cohen’s *d*
Father communication	9.88 (2.86)	9.45 (2.88)	7.85	<0.001	0.15
Mother communication	11.06 (2.76)	10.75 (2.83)	5.86	<0.001	0.11
Depressive symptoms	9.59 (3.57)	10.48 (3.78)	−12.96	<0.001	−0.24

**N_*Grade*7_ = 5,712, N_*Grade*9_ = 5,743.**

**TABLE 3:** Means and standard deviations of parent-adolescent communication and depressive symptoms according gender.

Variable	Boys	Girls	*t*	*p*	Cohen’s *d*
Father communication	9.66 (2.88)	9.66 (2.88)	0.10	0.924	0.00
Mother communication	10.51 (2.77)	11.28 (2.79)	−14.72	<0.001	−0.28
Depressive symptoms	9.82 (3.76)	10.26 (3.63)	−6.49	<0.001	−0.12

**N_*Boys*_ = 5,711, N_*Girls*_ = 5,744.**

**TABLE 4:** Means and standard deviations of parent-adolescent communication and depressive symptoms according gender and grade.

Gender	Variable	Grade 7	Grade 9	*t*	*p*	Cohen’s *d*
Boys	Father communication	9.87 (2.88)	9.45 (2.86)	5.32	<0.001	0.15
	Mother communication	10.69 (2.75)	10.32 (2.77)	5.00	<0.001	0.13
	Depressive symptoms	9.44 (3.61)	10.21 (3.86)	–7.73	<0.001	–0.21
Girls	Father communication	9.89 (2.85)	9.44 (2.90)	5.78	<0.001	0.16
	Mother communication	11.43 (2.73)	11.15 (2.83)	3.87	<0.001	0.10
	Depressive symptoms	9.75 (3.51)	10.74 (3.67)	–10.44	<0.001	–0.28

**N_*Boys*, Grade7_ = 2,921, N_*Boys*, Grade9_ = 2,790, N_*Girls*, Grade7_ = 2,791, N_*Girls*, Grade9_ = 2,953.**

### Effect of Parent-Adolescent Communication on Adolescents’ Depressive Symptoms

The results of the multiple regression analysis on the effect of parent-adolescent communication on depressive symptoms are provided in Table 5. Both father-adolescent communication and mother-adolescent communication had a significant negative effect on adolescents’ depressive symptoms, *B* = −0.121, *SE* = 0.015, *t* = −7.98, *p* < 0.001, 95% CI = [−0.150, −0.091] and *B* = −0.138, *SE* = 0.016, *t* = −8.59, *p* < 0.001, 95% CI = [−0.170, −0.107], respectively. Additionally, results indicated that adolescents in a rich family or middle income family had less depressive symptoms compared to adolescents in a poor family.

**TABLE 5:** Effect of father-adolescent and mother-adolescent communication on adolescents’ depressive symptoms.

Variable	Depressive symptoms				
	*B*	*SE*	*t*	*p*	95% CI
**School rank**					
Above average	0.080	0.101	0.79	0.428	−0.118, 0.279
Best	–0.063	0.125	–0.50	0.614	−0.307, 0.181
**School location**					
Urban-rural fringe area	–0.026	0.097	–0.27	0.791	−0.217, 0.165
Central urban area	0.280	0.099	2.83	0.005	0.086, 0.473
**Family economic status**					
Middle income	–0.712	0.094	–7.55	<0.001	−0.896, −0.527
Rich	–0.697	0.164	–4.26	<0.001	−1.018, −0.376
**Father education**	0.051	0.024	2.11	0.035	0.004, 0.097
**Mother education**	–0.082	0.024	–3.38	0.001	−0.129, −0.034
**Grade**	0.811	0.069	11.68	<0.001	0.675, 0.947
**Gender**	–0.514	0.070	–7.32	<0.001	−0.651, −0.376
**Father communication**	–0.121	0.015	–7.98	<0.001	−0.150, −0.091
**Mother communication**	–0.138	0.016	–8.59	<0.001	−0.170, −0.107

**CI, confidence interval. Grade: 0 = 7th grade, 1 = 9th grade. Gender: 0 = girl, 1 = boy. Reference group: Medium and below on School rank; town and country on School location; poor family on Family economic status.**

### Moderating Effects of Adolescents’ Age and Gender

#### Father-Adolescent Communication and Adolescents’ Depressive Symptoms

Table 6 displays the results of the multiple regression analysis on the moderating effects of age and gender in the relationship between father-adolescent communication and adolescents’ depressive symptoms. The result for the father communication × grade (age) × gender interaction variable suggests a trend toward significance at the 0.05 level (*B* = 0.075, *SE* = 0.048, *t* = 1.56, *p* = 0.119, 95% CI = [−019, 0.170]). Further analysis indicated that the moderating effect of grade (age) was significant for girls (*B* = −0.076, *SE* = 0.034, *t* = −2.241, *p* = 0.025, 95% CI = [−0.142, −0.010]), while the moderating effect was not significant for boys (*B* = 0.000, *SE* = 0.034, *t* = −0.014, *p* = 0.989, 95% CI = [−0.068, 0.067]). Simple effect analysis indicated that, for girls in grade 7, the effect of father-adolescent communication on depressive symptoms was significant (*B* = −0.096, *SE* = 0.026, *t* = −3.712, *p* < 0.001, 95% CI = [−0.147, −0.045]), but this effect was stronger for girls in grade 9 (*B* = −0.172, *SE* = 0.025, *t* = −6.906, *p* < 0.001, 95% CI = [−0.221, −0.123]). For boys in grade 7, the effect of father-adolescent communication on depressive symptoms was significant (*B* = −0.103, *SE* = 0.026, *t* = −3.975, *p* < 0.001, 95% CI = [−0.154, −0.052]), and this effect was similar for boys in grade 9 (*B* = −0.103, *SE* = 0.027, *t* = −3.892, *p* < 0.001, 95% CI = [−0.155, −0.051]).

**TABLE 6:** Moderating effect of adolescents’ grade and gender on the relation between father-adolescent communication and adolescents’ depressive symptoms.

Variable	Depressive symptoms				
	*B*	*SE*	*t*	*p*	95% CI
**School rank**					
Above average	0.079	0.101	0.78	0.436	−0.120, 0.278
Best	–0.063	0.125	–0.50	0.615	−0.307, 0.181
**School location**					
Urban-rural fringe area	–0.024	0.097	–0.249	0.803	−0.215, 0.167
Central urban area	0.283	0.099	2.860	0.004	0.089, 0.476
**Family economic status**					
Middle income	–0.708	0.094	–7.51	<0.001	−0.893, −0.523
Rich	–0.693	0.164	–4.23	<0.001	−1.014, −0.372
**Father education**	0.050	0.024	2.10	0.036	0.003, 0.097
**Mother education**	–0.081	0.024	–3.33	0.001	−0.128, −0.033
**Mother communication**	–0.139	0.016	–8.66	<0.001	−0.171, −0.108
**Father communication**	–0.119	0.015	–7.88	<0.001	−0.149, −0.089
**Grade**	0.811	0.069	11.69	<0.001	0.675, 0.947
**Gender**	–0.506	0.070	–7.20	<0.001	−0.644, −0.369
**Father communication × Grade**	–0.039	0.024	–1.61	0.108	−0.088, 0.009
**Father communication × Gender**	0.031	0.024	1.30	0.195	−0.016, 0.079
**Grade × Gender**	–0.136	0.139	–0.98	0.327	−0.407, 0.136
**Father communication × Grade × Gender**	0.075	0.048	1.56	0.119	−0.019, 0.170

**CI, confidence interval. Grade: 0 = 7th grade, 1 = 9th grade; Gender: 0 = girl, 1 = boy. *Reference group: Medium and below on School rank; town and country on School location; poor family on Family economic status.***

#### Mother-Adolescent Communication and Adolescents’ Depressive Symptoms

Table 7 displays the results of the multiple regression analysis on the moderating effects of age and gender in the relationship between mother-adolescent communication and adolescents’ depressive symptoms. The result for the mother communication × grade (age) × gender interaction variable was marginally significant (*B* = 0.086, *SE* = 0.051, *t* = 1.69, *p* = 0.090, 95% CI = [−0.014, 0.185]). This result indicates that the moderating effect of adolescents’ gender on the moderating effect of grade (age) on the relationship between mother-adolescent communication and adolescents’ depressive symptoms was significant. Further analysis revealed that the moderating effect of grade (age) was significant for girls (*B* = −0.075, *SE* = 0.035, *t* = −2.104, *p* = 0.035, 95% CI = [−0.144, −0.005]), while it was not significant for boys (*B* = 0.011, *SE* = 0.036, *t* = 0.310, *p* = 0.756, 95% CI = [−0.060, 0.082]). Simple effect analysis indicated that, for girls in grade 7, the effect of mother-adolescent communication on depressive symptoms was significant (*B* = −0.126, *SE* = 0.028, *t* = −4.593, *p* < 0.001, 95% CI = [−0.180, −0.072]), but this effect was stronger for girls in grade 9 (*B* = −0.201, *SE* = 0.026, *t* = −7.721, *p* < 0.001, 95% CI = [−0.252, −0.150]). For boys in grade 7, the effect of mother-adolescent communication on depressive symptoms was significant (*B* = −0.113, *SE* = 0.028, *t* = −4.096, *p* < 0.001, 95% CI = [−0.167, −0.059]), and this effect was similar for boys in grade 9 (*B* = −0.102, *SE* = 0.028, *t* = −3.658, *p* < 0.001, 95% CI = [−0.156, −0.047]).

**TABLE 7:** Moderating effect of adolescents’ grade and gender on the relation between mother-adolescent communication and adolescents’ depressive symptoms.

Variable	Depressive symptoms				
	*B*	*SE*	*t*	*p*	95% CI
**School rank**					
Above average	0.077	0.101	0.76	0.445	−0.121, 0.276
Best	–0.067	0.125	–0.54	0.591	−0.311, 0.177
**School location**					
Urban-rural fringe area	–0.023	0.097	–0.23	0.816	−0.213, 0.168
Central urban area	0.284	0.099	2.88	0.004	0.090, 0.478
**Family economic status**					
Middle income	–0.705	0.094	–7.48	< 0.001	−0.890, −0.520
Rich	–0.691	0.164	–4.22	< 0.001	−1.012, −0.370
**Father education**	0.049	0.024	2.06	0.039	0.002, 0.096
**Mother education**	–0.081	0.024	–3.33	0.001	−0.128, −0.033
**Father communication**	–0.122	0.015	–8.08	< 0.001	−0.152, −0.093
**Mother communication**	–0.136	0.016	–8.46	< 0.001	−0.168, −0.105
**Grade**	0.828	0.070	11.83	< 0.001	0.691, 0.966
**Gender**	–0.506	0.070	–7.20	< 0.001	−0.643, −0.368
**Mother communication × Grade**	–0.032	0.025	–1.28	0.201	−0.082, 0.017
**Mother communication × Gender**	0.057	0.025	2.23	0.026	0.007, 0.106
**Grade × Gender**	–0.154	0.140	–1.10	0.271	−0.427, 0.120
**Mother communication × Grade × Gender**	0.086	0.051	1.69	0.090	−0.014, 0.185

**CI, confidence interval. Grade: 0 = 7th grade, 1 = 9th grade; Gender: 0 = girl, 1 = boy. Reference group: Medium and below on School rank; town and country on School location; poor family on Family economic status.**

## Discussion

Our findings indicated that the quality of both father-adolescent and mother-adolescent communication was lower, while the level of adolescent depressive symptoms was higher among 9th grade students compared to 7th grade students. Moreover, the effects of both father-adolescent and mother-adolescent communication on adolescents’ depressive symptoms among all students were significant. Furthermore, gender and age jointly moderated the association between parental communication and adolescent depressive symptoms. Specifically, for girls, the negative effects of both father-adolescent and mother-adolescent communication on depressive symptoms were stronger in 9th grade students than in 7th grade students, while for boys, the negative effects did not differ between 7th grade students and 9th grade students. These findings suggest that the protective effects of positive parent-adolescent communication on adolescents’ depressive symptoms may be most salient among senior-grade girls in junior high school in China.

### Age Differences in Parent-Adolescent Communication and Adolescent Depressive Symptoms

When comparing 7th grade students (*M*_*age*_ = 13.14 years) to 9th grade students (*M*_*age*_ = 15.15 years), the results indicated that the quality of both father-adolescent and mother-adolescent communication was lower, while the level of adolescent depressive symptoms was higher among 9th grade students. This result supports Hypothesis 1, which is consistent with previous studies. Finkenauer et al. (2002) found that children were less likely to share their thoughts, feelings, and secrets with their parents when they got older, which is not beneficial for connectedness and intimacy between parents and their children (Metzger et al., 2012). Lionetti et al. (2019) examined the change in parent-adolescent communication using meta-analysis and found that parental control and adolescent disclosure decreased, but adolescents’ secrecy increased. In addition, Newman et al. (2007) found that 9th grade adolescents reported more depressive symptoms than 8th grade adolescents, and Chen et al. (2012) found that depressive symptoms increased from 18.8% in grade 7–21.7% in grade 9, based on a sample of 2,239 students from rural areas in China.

The finding regarding the positive association between age and depressive symptoms may be explained in part by the increase in academic stress that adolescents experience from grade 7 to 9. Ciciolla et al. (2017) found that children with poor academic performance were more likely to be depressed; in particular, maternal emphasis on high grades accompanied by parental criticism may lead to psychological maladjustment. Relative to children in primary school, Chinese adolescents experience higher levels of academic stress linked with high school entrance examinations due to limited educational resources, especially in grade 9 in middle school (Sun et al., 2012). As academic performance is highly valued in Chinese culture, studies have found that adolescents with poor academic grades were likely to be monitored strictly by parents, leading to more parental conflicts that increase the risk of depressive symptoms (Chao, 2001; Ma et al., 2018). Moreover, when adolescents considered parental monitoring as impaired independence and an invasion of privacy, they were more inclined to disclose to best friends rather than their parents (Metzger et al., 2012; Solis et al., 2015).

### Effects of Parent-Adolescent Communication on Adolescents’ Depressive Symptoms

We found that both father-adolescent and mother-adolescent communication negatively predicted adolescents’ depressive symptoms during junior high school, which supports Hypothesis 2. This result is consistent with previous findings showing that positive parental communication negatively related to adolescent depressive symptoms in Western cultures (Pinquart, 2017; Ioffe et al., 2020; Kapetanovic et al., 2020). Importantly, this result is consistent with a meta-analysis on risk factors associated with depressive symptoms among adolescents in mainland China (Tang et al., 2020) and highlights the protective effect of positive parent-adolescent communication on preventing depressive symptoms in adolescents in secondary schools in China.

Adolescents may face more stress from academic requirements and social relationships when entering middle school, which increases the risk of depressive symptoms (Verboom et al., 2013; Ma et al., 2018). However, according to Allen and Tan (2016), the attachment bond between adolescents and their parents could be maintained via positive parent-child communication, which, in turn, would help adolescents to deal with stress actively and make it less likely that they experience depressive symptoms. Studies have revealed that adolescents were more likely to communicate with parents who understand them well; as a result they get more guidance, involvement, and protection from their parents, which could prevent them from experiencing depressive symptoms (Kapetanovic et al., 2020; Hamza and Willoughby, 2011). However, if adolescents receive less support from parents by keeping secrets from them, they may experience more difficulty in relieving stress and reducing or alleviating depressive symptoms as a result (Flouri and Buchanan, 2003; Frijns et al., 2010). Ioffe et al. (2020) reported that positive parent-adolescent communication helped in developing problem-solving and adaptive coping skills to decrease the negative effects of stressful events in middle school students. Hill and Roberts (2019) suggested that positive parent-adolescent communication may increase self-efficacy and social skills that are helpful for academic success.

### The Moderating Effects of Adolescents’ Age and Gender

More importantly, we found that gender and age jointly moderated the association between parental communication and adolescent depressive symptoms. Specifically, for girls, the negative effects of both father-adolescent and mother-adolescent communication on depressive symptoms were stronger in 9th grade students than in 7th grade students, while for boys, the negative effects were similar among 9th grade students and 7th grade students. This finding was not consistent with Hypothesis 3. This result is also partially inconsistent with previous empirical evidence showing the protective effects of positive maternal communication on depressive symptoms for girls decreased during middle school but was not significant in boys (Ebbert et al., 2019). The different role expectations of fathers and mothers on boys and girls may explain these results. Chinese adolescents face more challenges in developing new interpersonal relationships and experience academic stress when they transition to middle school, which was found to be associated with depressive symptoms (Wang et al., 2016). Boys are expected to deal with these adjustment difficulties independently, whereas girls are expected to seek support from parents to solve them (Rosario et al., 1988; Cyranowski et al., 2000). Moreover, relative to boys, girls are more likely to express their emotional needs and share more information with their mothers (Collins and Russell, 1991; Kapetanovic et al., 2020). There is empirical evidence showing that girls received more problem-solving strategies and emotional support from parents than boys, which reduced the negative effects of depressive symptoms (Kenny et al., 2013; Van Lissa et al., 2019). When girls in the 9th grade experience more academic stress due to senior high school entrance examinations, they are more needed and likely to communicate with their parents for emotional and instrumental support than those in the 7th grade. Therefore, for girls, the effects of both father-adolescent and mother-adolescent communication on depressive symptoms may be stronger in the 9th grade relative to the 7th grade.

With regard to boys, those in the 9th grade require more autonomy and individuality than boys in the 7th grade, so they may become more secretive when relating with their parents in order to avoid interference in their development of individuality and autonomy from early adolescence to mid-adolescence (Keijsers et al., 2009). Chinese parents treat their sons with stricter rules and more behavior control compared to their daughters (Chuang and Su, 2009). Research indicates boys experienced more negative feelings when communicating with their parents, leading to less communication with their parents in order to avoid punishment (Shek, 2000; Rueger et al., 2014). Therefore, for boys, the negative effects of father-adolescent or mother-adolescent communication on adolescent depressive symptoms may be similar in both the 9th and 7th grades.

### Strengths and Limitations

This study makes important contributions to the literature by examining the combined moderating effects of adolescents’ age and gender on the association between parent-adolescent communication and early adolescents’ depressive symptoms. First, the present study replicated previous results that showed the protective effect of positive parent-adolescent communication on adolescent depressive symptoms (Tang et al., 2020) by using junior high school data from the China Education Panel Survey. Second, these findings contribute to acquiring knowledge in understanding the different protective effects of positive parent-adolescent communication on depressive symptoms among lower- and senior-grade female and male adolescents in junior high school in China.

However, the current study has several limitations. First, the present study was a cross-sectional survey. As such, causality between variables and the change of variables over time in this study could not be investigated. Second, self-report questionnaires were used to collect the data, which can influence the results due to response bias. Future studies should gather information from both parents and adolescents. Third, *ad hoc* questionnaires from the China Education Panel Survey were used, which may influence comparisons with other relevant research studies. Fourth, other possible moderators that might influence the association, e.g., parental stress, sensitivity, or mentalizing ability, were not controlled for. Finally, this study only investigated the effects of parent-driven communication, not child-driven communication, which might have different effects on adolescent depressive symptoms (Kapetanovic et al., 2020; Kapetanovic and Skoog, 2021). Further studies should examine the different roles of parent-driven and child-driven communication.

### Implications for Clinical Practice

The current findings provide key information for designing prevention and intervention programs to heighten the quality of parental communication with the aim of preventing or alleviating depressive symptoms in middle school children. As the findings identified age and gender differences, policymakers should provide more guidance and resources to encourage parents to communicate actively with their children, and clinicians should consider age and gender differences to meet the specific needs of girls and boys. Importantly, as the finding that positive parent-communication had a stronger protective effect on adolescent depressive symptoms among girls in the 9th grade relative to the 7th grade, parents should pay more attention to their daughters in the 9th grade and strengthen communication with them, which could effectively prevent or alleviate daughters’ depressive symptoms.

## Conclusion

Our study highlighted the importance of parent-adolescent communication in early adolescent depressive symptoms. We found a negative effect of both father-adolescent communication and mother-adolescent communication on girls’ depressive symptoms, which was enhanced in 9th grade girls compared to 7th grade girls during junior high school. In contrast, the negative effect of both father-adolescent communication and mother-adolescent communication on boys’ depressive symptoms did not differ between 7th grade and 9th grade during junior high school.

## Data Availability Statement

The raw data supporting the conclusions of this article will be available on request to the corresponding author.

## Ethics Statement

The studies involving human participants were reviewed and approved by the Research Ethics Committee of Renmin University of China. Written informed consent to participate in this study was provided by the participants’ legal guardian/next of kin.

## Author Contributions

YP and QZ were responsible for the design of the study, for describing the methods and results sections of the manuscript. QZ and HL were responsible for the introduction and discussion section of the manuscript. YP, QZ, and LZ were responsible for data analysis and revision of manuscript. All authors contributed with validating each other’s responsibilities and to the writing of the manuscript in its current presentation.

## Conflict of Interest

The authors declare that the research was conducted in the absence of any commercial or financial relationships that could be construed as a potential conflict of interest.
